# Supplementary material for: Comparison of Bacterial Community in the Jejunum, Ileum and Cecum of Suckling Lambs During Different Growth Stages
Source: Microorganisms. 2025 Aug 29;13(9):2024. doi: 10.3390/microorganisms13092024 (PMC12473106; doi:10.3390/microorganisms13092024)
Supplement: Supplementary file 1 [file microorganisms-13-02024-s001.zip › suppl.pdf]

Supplementary Materials

SUPPLEMENTARY TABLES

Table S1 Composition and nutrient levels of the starter (DM basis).

Ingredients, %	Content	Nutrient levels	Content
Corn	55.80	CP (%)	18.72
Soybean meal	20.20	EE (%)	5.40
Cottonseed mean	18.70	NDF (%)	21.67
NaCl	0.30	ADF (%)	8.11
$NaHCO_3$	0.60	Ca (%)	0.78
$CaHPO_4$	0.40	P (%)	0.41
Premix[1]	4.00	ME[2] (MJ/kg)	17.62

DM, dry matter; CP, crude protein; EE, ether extract; NDF, neutral detergent fiber; ADF, acid detergent fiber; ME, metabolizable energy.

[1] The premix provided following per kilogram of diet: Fe 280 mg, Zn 50 mg, Mn 40 mg, Cu 10 mg, I 0.60 mg, Se 0.40 mg, Co 0.20 mg, VA 8000 IU, VD 900 IU, VE 30 IU.

[2] ME was a calculated value; the other nutritional levels were measured values.

Table S2 Data acquisition of all samples.

Items	Simple no.	Raw sequences	Effective sequences	OTUs
Jejunum	J_D0_1	32575	31878	1457
	J_D0_2	38567	37648	1561
	J_D0_3	34445	33709	1471
	J_D0_4	31806	30987	1473
	J_D0_5	32694	31944	1512
	J_D7_1	37599	36682	227
	J_D7_2	30907	30096	164
	J_D7_3	33479	32611	175
	J_D7_4	32501	31856	155
	J_D7_5	39963	38995	235
	J_D28_1	36919	36075	775

	J_D28_2	35711	34927	577
	J_D28_3	34594	33852	577
	J_D28_4	34279	33645	723
	J_D28_5	30619	29924	511
Ileum	I_D0_1	38321	37618	407
	I_D0_2	38222	37363	274
	I_D0_3	39768	38999	122
	I_D0_4	30414	29789	178
	I_D0_5	37151	36413	198
	I_D7_1	32258	31636	79
	I_D7_2	35189	33963	267
	I_D7_3	32242	31467	312
	I_D7_4	32830	32187	369
	I_D7_5	35041	34309	317
	I_D28_1	33095	32367	152
	I_D28_2	35134	34309	123
	I_D28_3	36839	35926	151
	I_D28_4	38175	37386	146
	I_D28_5	38712	37783	172
Cecum	C_D0_1	35062	34431	98
	C_D0_2	32431	31841	174
	C_D0_3	33858	33191	283
	C_D0_4	32979	32300	391
	C_D0_5	34120	33354	326
	C_D7_1	31025	30410	143
	C_D7_2	34607	33857	89
	C_D7_3	36542	35875	134
	C_D7_4	36685	35947	142
	C_D7_5	39795	38981	112
	C_D28_1	30885	30152	187
	C_D28_2	34911	34203	172
	C_D28_3	32652	31854	197
	C_D28_4	35757	34945	459
	C_D28_5	30195	29522	295

Table S3 Analysis of permutational multivariate analysis of variance results of bacterial community according to the intestinal region of lambs in three different age groups.

**Groups**	**r.value**	***P*-value**
J_D0 vs J_D7	1	0.009
J_D0 vs J_D28	1	0.011
J_D7 vs J_D28	1	0.007

J_D0 vs J_D7 vs J_D28	1	0.001
I_D0 vs I_D7	1	0.01
I_D0 vs I_D28	0.8	0.012
I_D7 vs I_D28	0.326	0.051
I_D0 vs I_D7 vs I_D28	0.752	0.001
C_D0 vs C_D7	0.076	0.24
C_D0 vs C_D28	1	0.005
C_D7 vs C_D28	0.64	0.013
C_D0 vs C_D7 vs C_D28	0.551	0.001
J_D0 vs I_D0	1	0.007
J_D0 vs C_D0	1	0.014
I_D0 vs C_D0	0.596	0.009
J_D0 vs I_D0 vs C_D0	0.901	0.001
J_D7 vs I_D7	1	0.006
J_D7 vs C_D7	0.696	0.007
I_D7 vs C_D7	0.58	0.008
J_D7 vs I_D7 vs C_D7	0.706	0.001
J_D28 vs I_D28	1	0.012
J_D28 vs C_D28	1	0.007
I_D28 vs C_D28	0.192	0.075
J_D28 vs I_D28 vs C_D28	0.82	0.001

Table S4 Comparison of the phylum across the intestinal region of lambs (The criterion of average relative abundance ≥1% in at least one age group within a certain intestinal region).

Items	0d	7d	28d	*P*-value
Jejunum				
Firmicutes	26.5 ± 1.65^{b}	83.68 ± 0.49^{a}	30.58 ± 1.82^{b}	< 0.001
Proteobacteria	26.56 ± 0.45^{b}	0.32 ± 0.02^{c}	42.16 ± 1.3^{a}	< 0.001
Bacteroidetes	19.78 ± 0.76^{b}	0.36 ± 0.02^{c}	21.99 ± 0.91^{a}	< 0.001
Actinobacteria	5.95 ± 0.22^{b}	10.36 ± 0.27^{a}	2.67 ± 0.73^{c}	< 0.001
Chloroflexi	6.05 ± 0.18^{a}	0.05 ± 0.02^{b}	0.08 ± 0.02^{b}	< 0.001
Acidobacteria	5.54 ± 0.35^{a}	0.02 ± 0.01^{b}	0.05 ± 0.01^{b}	< 0.001
Epsilonbacteraeota	0.92 ± 0.04^{b}	0^{c}	1.48 ± 0.06^{a}	< 0.001
Spirochaetes	2.19 ± 0.07^{a}	0^{b}	0.03 ± 0.01^{b}	< 0.001
Planctomycetes	1.56 ± 0.1^{a}	0.01 ± 0.01^{b}	0.01 ± 0.003^{b}	< 0.001
Verrucomicrobia	1.27 ± 0.08^{a}	0 ± 0.002^{b}	0.01 ± 0.003^{b}	< 0.001
Others	3.68 ± 0.15^{b}	5.18 ± 0.56^{a}	0.94 ± 0.06^{c}	< 0.001
Ileum				
Firmicutes	57.4 ± 9.17^{b}	94.09 ± 1.41^{a}	87.9 ± 4.57^{a}	0.002
Proteobacteria	40.86 ± 9.56^{a}	2.82 ± 1.18^{b}	0.91 ± 0.38^{b}	< 0.001
Actinobacteria	0.5 ± 0.13	1.04 ± 0.33	9.38 ± 4.7	0.070

Bacteroidetes	0.94 ± 0.22	1.25 ± 0.52	0.38 ± 0.13	0.213
Others	0.31 ± 0.05	0.8 ± 0.28	1.43 ± 0.72	0.240
Cecum				
Firmicutes	53.46 ± 14.05	61.24 ± 16.33	87.29 ± 2.17	0.177
Proteobacteria	44.32 ± 14.45	37.52 ± 16.38	0.36 ± 0.06	0.063
Actinobacteria	1.23 ± 0.3[b]	0.61 ± 0.2[b]	8.03 ± 2.47[a]	0.006
Bacteroidetes	0.58 ± 0.12	0.41 ± 0.07	2.13 ± 1.66	0.407
Verrucomicrobia	0.02 ± 0.01	0.0007 ± 0.0007	0.8 ± 0.56	0.173
Tenericutes	0.01 ± 0.004	0.002 ± 0.001	0.31 ± 0.2	0.129
Planctomycetes	0.03 ± 0.01	0.01 ± 0.2	0.21 ± 0.07	0.440
Others	0.36 ± 0.07	0.19 ± 0.03	0.86 ± 0.33	0.082

Table S5 Comparison of the phylum across the intestinal region of lambs (The criterion of average relative abundance ≥1% in at least one intestinal region within a certain age group).

**Items**	**Je**	**Il**	**Ce**	***P*-value**
0d				
Firmicutes	26.36 ± 1.6	57.66 ± 9.2	53.64 ± 14.2	0.087
Proteobacteria	26.29 ± 0.36	40.6 ± 9.58	44.12 ± 14.58	0.440
Bacteroidetes	19.95 ± 0.78[a]	0.95 ± 0.24[b]	0.56 ± 0.11[b]	< 0.001
Actinobacteria	5.95 ± 0.29[a]	0.44 ± 0.11[c]	1.26 ± 0.31[b]	< 0.001
Chloroflexi	6.06 ± 0.24[a]	0.1 ± 0.02[b]	0.09 ± 0.03[b]	< 0.001
Acidobacteria	5.62 ± 0.3[a]	0.09 ± 0.01[b]	0.1 ± 0.02[b]	< 0.001
Spirochaetes	2.17 ± 0.06[a]	0.005 ± 0.002[b]	0.005 ± 0.002[b]	< 0.001
Planctomycetes	1.58 ± 0.07[a]	0.01 ± 0.01[b]	0.02 ± 0.004[b]	< 0.001
Verrucomicrobia	1.31 ± 0.07[a]	0.02 ± 0.01[b]	0.02 ± 0.003[b]	< 0.001
Epsilonbacteraeota	1 ± 0.07[a]	0.03 ± 0.01[b]	0.03 ± 0.01[b]	< 0.001
Others	3.71 ± 0.09[a]	0.1 ± 0.02[b]	0.15 ± 0.03[b]	< 0.001
7d				
Firmicutes	83.67 ± 0.44	94.21 ± 1.38	61.38 ± 16.36	0.081
Proteobacteria	0.34 ± 0.02[b]	2.84 ± 1.15[b]	37.47 ± 16.41[a]	0.030
Actinobacteria	10.38 ± 0.37[a]	1 ± 0.32[b]	0.6 ± 0.2[b]	< 0.001
Bacteroidetes	0.32 ± 0.02	1.14 ± 0.46	0.36 ± 0.07	0.089
Others	5.24 ± 0.54[a]	0.67 ± 0.29[b]	0.16 ± 0.03[b]	< 0.001
28d				
Firmicutes	30.95 ± 2[b]	87.5 ± 4.71[a]	87.26 ± 2.13[a]	< 0.001
Proteobacteria	41.94 ± 1.47[a]	1.01 ± 0.41[b]	0.39 ± 0.07[b]	< 0.001
Bacteroidetes	21.78 ± 0.85[a]	0.41 ± 0.14[b]	2.05 ± 1.56[b]	< 0.001
Actinobacteria	2.64 ± 0.64	9.52 ± 4.81	8.12 ± 2.53	0.302
Epsilonbacteraeota	1.53 ± 0.07[a]	0.01 ± 0.002[b]	0.01 ± 0.01[b]	< 0.001
Verrucomicrobia	0.01 ± 0.01	0.01 ± 0.01	0.83 ± 0.58	0.173
Tenericutes	0.02 ± 0.004	0	0.31 ± 0.19	0.141

Others	1.14 ± 0.09	1.55 ± 0.85	1.03 ± 0.33	0.767

Table S6 Comparison of the genus across the intestinal region of lambs (The criterion of average relative abundance ≥1% in at least one age group within a certain intestinal region).

**Items**		**0d**	**7d**	**28d**	***P*-value**
Jejunum					
Firmicutes	Lactobacillus	4.66 ± 0.71^{b}	23.4 ± 0.2^{a}	5.16 ± 1.68^{b}	< 0.001
	Lachnospiraceae NK3A20 group	0.07 ± 0.02^{b}	27.89 ± 0.65^{a}	0.51 ± 0.2^{b}	< 0.001
	Acetitomaculum	0.03 ± 0.01^{c}	10.59 ± 0.11^{a}	0.33 ± 0.12^{b}	< 0.001
	Roseburia	0.82 ± 0.08^{c}	4.26 ± 0.16^{a}	2.57 ± 0.13^{b}	< 0.001
	Weissella	0.51 ± 0.18^{b}	0.01 ± 0.01^{c}	4.65 ± 0.15^{a}	< 0.001
	[Eubacterium] coprostanoligenes group	0.31 ± 0.08^{b}	4.06 ± 0.15^{a}	0.21 ± 0.1^{b}	< 0.001
	[Ruminococcus] gauvreauii group	0.03 ± 0.01^{b}	4.26 ± 0.14^{a}	0.16 ± 0.06^{b}	< 0.001
	Unclassified Lachnospiraceae	1.16 ± 0.08^{b}	0.32 ± 0.12^{c}	2.78 ± 0.15^{a}	< 0.001
	Streptococcus	2.42 ± 0.17^{a}	0.01 ± 0.004^{c}	0.56 ± 0.03^{b}	< 0.001
Proteobacteria	Ralstonia	1.73 ± 0.08^{b}	0.03 ± 0.01^{c}	21.63 ± 0.54^{a}	< 0.001
	Pseudomonas	1.23 ± 0.29^{b}	0.01 ± 0.01^{c}	7.42 ± 0.41^{a}	< 0.001
	Escherichia-Shigella	6.63 ± 0.11^{a}	0.12 ± 0.01^{c}	1.21 ± 0.04^{b}	< 0.001
	Psychrobacter	0.28 ± 0.11^{b}	0^{b}	2.43 ± 0.16^{a}	< 0.001
Bacteroidetes	Unclassified Muribaculaceae	6.19 ± 0.22^{b}	0.08 ± 0.01^{c}	8.78 ± 0.42^{a}	< 0.001
	Bacteroides	3.09 ± 0.16^{b}	0.05 ± 0.01^{c}	3.95 ± 0.19^{a}	< 0.001
	Alistipes	1.56 ± 0.16^{b}	0.09 ± 0.02^{c}	3.21 ±	< 0.001

	Chryseobacterium	2.29 ± 0.2[a]	0.02 ± 0.004[b]	0.16[a] 0.24 ± 0.06[b]	< 0.001
Actinobacteria	Olsenella	0.05 ± 0.01[c]	6.85 ± 0.16[a]	0.82 ± 0.31[b]	< 0.001
	Bifidobacterium	1.25 ± 0.18	1.15 ± 0.04	1 ± 0.48	0.830
Ileum					
Firmicutes	Lactobacillus	46.96 ± 10.27[b]	79.61 ± 9[a]	74.22 ± 5.91[a]	0.043
	Enterococcus	6.66 ± 1.5	4.02 ± 2.38	0.31 ± 0.14	0.051
	Butyricicoccus	0.1 ± 0.02	6.01 ± 3.9	0.02 ± 0.01	0.139
	Lachnospiraceae NK3A20 group	0.13 ± 0.01	0.13 ± 0.02	2.32 ± 1.35	0.114
	Acetitomaculum	0.04 ± 0.01	0.02 ± 0.01	1.74 ± 0.88	0.053
	Veillonella	0.04 ± 0.01	1.44 ± 0.89	0.02 ± 0.01	0.123
	Turicibacter	0.01 ± 0.01	0.02 ± 0.004	1.37 ± 0.85	0.117
	Clostridium sensu stricto 1	0.03 ± 0.003	0.03 ± 0.01	1.09 ± 0.69	0.135
	[Ruminococcus] gauvreauii group	0.02 ± 0.01	0.02 ± 0.01	1 ± 0.75	0.222
	[Eubacterium] nodatum group	0.004 ± 0.002	0.01 ± 0.004	0.9 ± 0.61	0.157
	[Eubacterium] coprostanoligenes group	0.05 ± 0.01	0.03 ± 0.01	0.59 ± 0.52	0.355
Proteobacteria	Escherichia-Shigella	29.35 ± 7.09[a]	2.01 ± 1.15[b]	0.59 ± 0.31[b]	0.001
	Mannheimia	7.59 ± 1.84[a]	0.01 ± 0.01[b]	0[b]	< 0.001
	Enterobacter	2.11 ± 0.44 [a]	0.02 ± 0.01 [b]	0.02 ± 0.01[b]	0.001
Actinobacteria	Olsenella	0.05 ± 0.004	0.07 ± 0.02	6.79 ± 4.26	0.125
	Bifidobacterium	2.11 ± 0.44	0.02 ± 0.01	0.02 ± 0.01	0.246
Cecum					
Firmicutes	Lactobacillus	3.89 ± 1.2	35.47 ± 19.46	37.23 ±	0.126

			6.78	
Butyricicoccus	11.95 ± 4.02	4.18 ± 3.72	0.07 ± 0.04	0.058
Blautia	1.16 ± 0.47^{b}	1.66 ± 1.38^{b}	9.57 ± 3.39^{a}	0.028
Sellimonas	10.14 ± 4.14 ^{a}	1.67 ± 0.73 ^{b}	0.04 ± 0.02 ^{b}	0.026
Enterococcus	0.88 ± 0.54	7.83 ± 4.49	0.06 ± 0.002	0.109
[Eubacterium] coprostanoligenes group	2.62 ± 1.06	0.22 ± 0.15	3.56 ± 1.99	0.215
Caproiciproducens	3.65 ± 1.49	2.43 ± 1.24	0.004 ± 0.004	0.103
Clostridium sensu stricto 1	5.2 ± 3.22	0.36 ± 0.22	0.03 ± 0.01	0.133
Lachnospiraceae NK3A20 group	0.2 ± 0.03	0.13 ± 0.01	4.28 ± 3.96	0.369
Lachnoclostridium	1.94 ± 0.78	0.99 ± 0.5	1.63 ± 0.32	0.501
[Ruminococcus] torques group	2.37 ± 0.97	0.14 ± 0.09	1.74 ± 0.86	0.136
Ruminococcaceae UCG-005	0.01 ± 0.004	0.01 ± 0.01	3.57 ± 1.87	0.058
Ruminococcaceae UCG-014	0.06 ± 0.02^{b}	0.6 ± 0.59^{b}	2.67 ± 0.72^{a}	0.012
GCA-900066225	1.38 ± 0.57	1.76 ± 1.19	0.05 ± 0.03	0.285
Christensenellaceae R-7 group	0.03 ± 0.01	0	2.93 ± 1.77	0.106
Anaerotruncus	1.97 ± 0.81	0.79 ± 0.4	0.01 ± 0.01	0.060
Hydrogenoanaerobacterium	2 ± 0.82^{a}	0.52 ± 0.34^{ab}	0.01 ± 0.01^{b}	0.046
Marvinbryantia	0.001 ± 0.001^{b}	0.01 ± 0.01^{b}	1.45 ± 0.61^{a}	0.019
Unclassified Lachnospiraceae	0.15 ± 0.06	0.04 ± 0.01	1.27 ± 0.69	0.095
[Ruminococcus] gauvreauii group	0.04 ± 0.01^{b}	0.03 ± 0.01^{b}	1.35 ± 0.61^{a}	0.032
Unclassified	0.51 ± 0.21	0.09 ± 0.06	0.72 ±	0.549

	Ruminococcaceae			0.67	
	Ruminococcus 2	0.06 ± 0.03	0.17 ± 0.17	1.02 ± 0.55	0.127
	Turicibacter	0.01 ± 0.004	0.28 ± 0.12	0.9 ± 0.57	0.206
	Phascolarctobacterium	0.01 ± 0.004	0.002 ± 0.002	0.88 ± 0.79	0.328
Proteobacteria	Escherichia-Shigella	32.57 ± 8.28	36.67 ± 16.24	0.11 ± 0.01	0.059
	Enterobacter	7.22 ± 4.36	0.1 ± 0.04	0.01 ± 0.01	0.107
	Citrobacter	2.23 ± 1.37	0	0	0.110
Actinobacteria	Olsenella	0.05 ± 0.02[b]	0.02 ± 0.01[b]	4.05 ± 1.52[a]	0.010
	Bifidobacterium	0.03 ± 0.01	0.03 ± 0.02	2.8 ± 1.51	0.070
Bacteroidetes	Prevotellaceae NK3B31 group	0.002 ± 0.002	0	1.01 ± 1.01	0.394
Verrucomicrobia	Akkermansia	0.002 ± 0.002	0	0.8 ± 0.56	0.171

Table S7 Comparison of the genus across the intestinal region of lambs (The criterion of average relative abundance ≥2.5% in at least one intestinal region within a certain age group).

**Items**		**Je**	**Il**	**Ce**	***P*-value**
0d					
Firmicutes	Lactobacillus	4.54 ± 0.68[b]	47.21 ± 10.34[a]	3.97 ± 1.23[b]	< 0.001
	Butyricicoccus	0.08 ± 0.01[b]	0.1 ± 0.02[b]	12.06 ± 4.05[a]	0.005
	Sellimonas	0.01 ± 0.01[b]	0.35 ± 0.08[b]	10.07 ± 4.11[a]	0.017
	Enterococcus	0.84 ± 0.05[b]	6.58 ± 1.55[a]	0.9 ± 0.54[b]	0.001
	Clostridium sensu stricto 1	0.82 ± 0.1	0.03 ± 0.004	5.1 ± 3.16	0.150
	Caproiciproducens	0.02 ± 0.004[b]	0.31 ± 0.07[b]	3.57 ± 1.46[a]	0.020
	Streptococcus	2.36 ± 0.19[a]	0.3 ± 0.1[b]	0.36 ± 0.09[b]	< 0.001
	[Eubacterium] coprostanoligenes group	0.3 ± 0.08[b]	0.07 ± 0.02[b]	2.64 ± 1.06[a]	< 0.001
	[Ruminococcus]	0.22 ± 0.01[b]	0.05 ± 0.02[b]	2.35 ± 0.96[a]	0.022

	torques group				
	Lachnoclostridium	0.28 ± 0.03[b]	0.38 ± 0.13[b]	1.89 ± 0.77[a]	0.047
	Anaerotruncus	0.02 ± 0.01[b]	0.05 ± 0.01[b]	2.09 ± 0.86[a]	0.018
	Hydrogenoanaerobacterium	0[b]	0.04 ± 0.01[b]	2.01 ± 0.83[a]	0.017
	GCA-900066225	0.01 ± 0.01[b]	0.41 ± 0.1[ab]	1.45 ± 0.59[a]	0.033
Proteobacteria	Escherichia-Shigella	6.57 ± 0.13[b]	29.15 ± 7.14[a]	32.31 ± 8.34[b]	0.028
	Enterobacter	1.37 ± 0.11	2.08 ± 0.46	7.22 ± 4.36	0.244
	Mannheimia	0[b]	7.54 ± 1.78[a]	0.03 ± 0.02[b]	< 0.001
	Citrobacter	0	0.004 ± 0.004	2.33 ± 1.43	0.111
Bacteroidetes	Unclassified Muribaculaceae	6.19 ± 0.23[a]	0.16 ± 0.05[b]	0.08 ± 0.03[b]	< 0.001
	Bacteroides	3.05 ± 0.17[a]	0.07 ± 0.02[b]	0.07 ± 0.02[b]	< 0.001
	Chryseobacterium	2.28 ± 0.2[a]	0.39 ± 0.09[b]	0.08 ± 0.03[b]	< 0.001
7d					
Firmicutes	Lactobacillus	24.01 ± 0.22[b]	79.64 ± 9.07[a]	35.61 ± 19.59[b]	0.020
	Lachnospiraceae NK3A20 group	27.6 ± 0.63[a]	0.12 ± 0.01[b]	0.12 ± 0.01[b]	< 0.001
	Enterococcus	0.05 ± 0.02	4.12 ± 2.43	7.93 ± 4.52	0.213
	Acetitomaculum	10.63 ± 0.22[a]	0.01 ± 0.01[b]	0.05 ± 0.03[b]	< 0.001
	Butyricicoccus	0.06 ± 0.02	5.93 ± 3.82	4.17 ± 3.7	0.410
	[Eubacterium] coprostanoligenes group	4.09 ± 0.12[a]	0.03 ± 0.01[b]	0.19 ± 0.13[b]	< 0.001
	[Ruminococcus] gauvreauii group	4.25 ± 0.1[a]	0.02 ± 0.01[b]	0.02 ± 0.01[b]	< 0.001
	Roseburia	4.2 ± 0.2[a]	0.03 ± 0.01[b]	0.01 ± 0.01[b]	< 0.001
	Caproiciproducens	0.02 ± 0.01	0.8 ± 0.5	2.34 ± 1.22	0.132
	Blautia	0.14 ± 0.02	0.21 ± 0.16	1.68 ± 1.4	0.349
	GCA-900066225	0.01 ± 0.004	0.21 ± 0.14	1.74 ± 1.2	0.202
	Sellimonas	0.03 ± 0.01[b]	0.001 ± 0.001[b]	1.67 ± 0.74[a]	0.026
	Lachnoclostridium	0.02 ± 0.02	0.51 ± 0.23	1.05 ± 0.54	0.142
	Veillonella	0 ± 0	1.45 ± 0.92	0.02 ± 0.01	0.128
	Ruminococcaceae UCG-014	0.19 ± 0.03	0.03 ± 0.02	0.56 ± 0.53	0.494
Proteobacteria	Escherichia-Shigella	0.12 ± 0.02[b]	1.97 ± 1.13[b]	36.63 ± 16.27[a]	0.030
Actinobacteria	Olsenella	6.86 ± 0.19[a]	0.07 ± 0.02[b]	0.03 ± 0.01[b]	< 0.001

28d					
Firmicutes	Lactobacillus	5.37 ± 1.81[c]	73.69 ± 5.89[a]	36.7 ± 6.66[b]	< 0.001
	Blautia	0.17 ± 0.01[b]	0.1 ± 0.06[b]	9.46 ± 3.31[a]	0.006
	Lachnospiraceae NK3A20 group	0.56 ± 0.21	2.45 ± 1.36	4.79 ± 4.49	0.558
	Weissella	4.67 ± 0.14[a]	0.02 ± 0.01[b]	0.01 ± 0.01[b]	< 0.001
	[Eubacterium] coprostanoligenes group	0.22 ± 0.1	0.63 ± 0.54	3.57 ± 2.02	0.144
	Unclassified Lachnospiraceaea	2.81 ± 0.12[a]	0.07 ± 0.03[b]	1.22 ± 0.68[b]	0.001
	Ruminococcaceae UCG-005	0.04 ± 0.01	0.01 ± 0.003	3.47 ± 1.88	0.069
	Ruminococcaceae UCG-014	0.32 ± 0.05[b]	0.09 ± 0.02[b]	2.65 ± 0.72[a]	0.002
	Christensenellaceae R-7 group	0.05 ± 0.01	0.01 ± 0.01	2.92 ± 1.76	0.107
	Roseburia	2.64 ± 0.12[a]	0.1 ± 0.03[b]	0.08 ± 0.04[b]	< 0.001
	[Ruminococcus] gauvreauii group	0.17 ± 0.05	1.08 ± 0.78	1.39 ± 0.7	0.364
	Acetitomaculum	0.31 ± 0.11	1.53 ± 0.72	0.78 ± 0.3	0.203
	Turicibacter	0.01 ± 0.01	1.4 ± 0.87	0.89 ± 0.57	0.293
	Ruminococcus 2	0.32 ± 0.2	0.71 ± 0.48	1.08 ± 0.61	0.534
	[Eubacterium] nodatum group	0.14 ± 0.1	0.87 ± 0.6	0.86 ± 0.35	0.381
	Marvinbryantia	0.02 ± 0.01[b]	0[b]	1.44 ± 0.61[a]	0.020
	Clostridium sensu stricto 1	0.2 ± 0.03	1.13 ± 0.72	0.05 ± 0.02	0.175
	Phascolarctobacterium	0.2 ± 0.03	0.02 ± 0.01	0.84 ± 0.75	0.401
	Unclassified Ruminococcaceae	0.21 ± 0.04	0.001 ± 0.001	0.75 ± 0.69	0.420
Proteobacteria	Ralstonia	21.64 ± 0.66[a]	0.04 ± 0.01[b]	0.02 ± 0.01[b]	< 0.001
	Pseudomonas	7.35 ± 0.44[a]	0.02 ± 0.01[b]	0.01 ± 0.01[b]	< 0.001
	Psychrobacter	2.37 ± 0.18[a]	0[b]	0.002 ± 0.02[b]	< 0.001
Actinobacteria	Olsenella	0.8 ± 0.28	6.87 ± 4.38	4.03 ± 1.54	0.313
	Bifidobacterium	1 ± 0.46	2.14 ± 1.7	2.89 ± 1.56	0.623
Bacteroidetes	Unclassified Muribaculaceae	8.71 ± 0.33[b]	0.08 ± 0.03[b]	0.04 ± 0.01[a]	< 0.001
	Bacteroides	3.71 ± 0.14[a]	0.01 ±	0.27 ± 0.15[b]	< 0.001

			0.004[b]		
	Alistipes	3.21 ± 0.14[a]	0.07 ± 0.01[b]	0.32 ± 0.19[b]	< 0.001
	Prevotellaceae NK3B31 group	0.05 ± 0.01	0.001 ± 0.002	0.92 ± 0.92	0.414
Verrucomicrobia	Akkermansia	0.002 ± 0.002	0	0.83 ± 0.58	0.172

Table S8 Predicted functions on the level 1 of the digesta-associated bacteria throughout the intestinal region of lambs.

**Items**	**Age in days**	**Je**	**Il**	**Ce**	***P*-value**
Cellular Processes	0	16.69 ± 0.04	16.37 ± 1.22	19.11 ± 0.84	0.084
	7	13.27 ± 0.04[a]	8.55 ± 0.98[b]	16.58 ± 2.19[a]	0.005
	28	18.77 ± 0.18[a]	9.40 ± 0.56[c]	12.72 ± 0.77[b]	< 0.001
Environmental Information Processing	0	10.84 ± 0.07[c]	14.56 ± 0.45[a]	14.48 ± 0.64[a]	< 0.001
	7	10.07 ± 0.03[b]	9.87 ± 0.39[b]	13.79 ± 0.75[a]	< 0.001
	28	12.05 ± 0.06[a]	10.50 ± 0.26[b]	10.54 ± 0.19[b]	< 0.001
Genetic Information Processing	0	31.20 ± 0.09	31.45 ± 2.45	27.36 ± 1.92	0.236
	7	37.38 ± 0.08	39.35 ± 0.83	30.23 ± 4.28	0.058
	28	25.89 ± 0.36[c]	41.37 ± 0.41[a]	38.55 ± 0.60[b]	< 0.001
Human Diseases	0	11.95 ± 0.03	12.38 ± 0.20	12.06 ± 0.47	0.585
	7	11.06 ± 0.01[b]	12.69 ± 0.18[a]	13.19 ± 0.43[a]	< 0.001
	28	14.53 ± 0.07[a]	11.88 ± 0.32[b]	11.01 ± 0.10[c]	< 0.001
Metabolism	0	16.56 ± 0.04[a]	15.27 ± 0.23[c]	16.03 ± 0.03[b]	< 0.001
	7	15.65 ± 0.01[a]	16.15 ± 0.15[a]	14.71 ± 0.35[b]	0.002
	28	15.74 ± 0.02	15.72 ± 0.26	15.82 ± 0.11	0.905
Organismal Systems	0	12.75 ± 0.06[a]	9.96 ± 0.36[c]	10.97 ± 0.06[b]	< 0.001
	7	12.57 ± 0.03[ab]	13.39 ± 0.24[a]	11.49 ± 0.69[b]	0.024
	28	13.03 ± 0.05[a]	11.12 ± 0.31[b]	11.36 ± 0.12[b]	< 0.001

Table S9 Predicted functions on the level 2 of the digesta-associated bacteria throughout the intestinal region of lambs (The criterion that relative abundance is ≥5% in at least one intestinal region during any age group).

**Items**		**Je**	**Il**	**Ce**	***P*-value**
Aging	0	7.24 ± 0.05[a]	5.72 ± 0.37[b]	6.02 ± 0.47[b]	0.021
	7	4.00 ± 0.02[b]	3.73 ± 0.10[b]	5.27 ± 0.66[a]	0.034
	28	6.14 ± 0.06[a]	3.24 ± 0.05[b]	3.49 ± 0.12[b]	< 0.001
Cell motility	0	3.98 ± 0.03	4.45 ± 0.77	5.84 ± 0.37	0.052
	7	2.82 ± 0.03	1.08 ± 0.55	4.38 ± 1.38	0.056
	28	4.64 ± 0.07[a]	0.80 ± 0.11[c]	2.48 ± 0.56[b]	< 0.001
Drug resistance:	0	6.93 ± 0.01[a]	6.99 ± 0.07[a]	6.46 ± 0.01[b]	< 0.001

antineoplastic	7	6.22 ± 0.01^{b}	7.21 ± 0.15^{a}	6.14 ± 0.16^{b}	< 0.001
	28	7.61 ± 0.02^{a}	6.98 ± 0.22^{b}	6.57 ± 0.10^{b}	0.001
Folding, sorting and degradation	0	4.30 ± 0.01	4.57 ± 0.17	4.31 ± 0.18	0.379
	7	5.15 ± 0.01	4.64 ± 0.05	4.61 ± 0.32	0.115
	28	3.46 ± 0.04^{b}	5.09 ± 0.12^{a}	5.10 ± 0.02^{a}	< 0.001
Nucleotide metabolism	0	3.49 ± 0.01^{b}	4.22 ± 0.15^{a}	3.76 ± 0.06^{b}	0.001
	7	4.33 ± 0.002^{b}	5.18 ± 0.15^{a}	4.05 ± 0.29^{b}	0.003
	28	3.01 ± 0.03^{c}	5.06 ± 0.18^{a}	4.50 ± 0.11^{b}	< 0.001
Replication and repair	0	5.18 ± 0.03	5.99 ± 0.57	5.08 ± 0.39	0.242
	7	6.43 ± 0.01	6.83 ± 0.09	5.63 ± 0.97	0.341
	28	4.07 ± 0.07^{c}	7.41 ± 0.13^{a}	6.88 ± 0.10^{b}	< 0.001
Translation	0	4.74 ± 0.02^{cd}	5.15 ± 0.57^{bc}	4.20 ± 0.41^{cd}	0.289
	7	6.23 ± 0.02	6.21 ± 0.11	4.83 ± 0.96	0.168
	28	3.71 ± 0.07^{c}	6.99 ± 0.12^{a}	6.48 ± 0.12^{b}	< 0.001
